# Iodine and Iodine Deficiency: A Comprehensive Review of a Re-Emerging Issue

**DOI:** 10.3390/nu14173474

**Published:** 2022-08-24

**Authors:** Adrienne Hatch-McChesney, Harris R. Lieberman

**Affiliations:** Military Nutrition Division, U.S. Army Research Institute of Environmental Medicine, 10 General Greene Ave, Natick, MA 01760, USA

**Keywords:** iodine deficiency disorders, urinary iodine concentration, thyroid stimulating hormone, iodized salt, supplementation

## Abstract

Iodine is a mineral nutrient essential for the regulation of a variety of key physiological functions including metabolism and brain development and function in children and adults. As such, iodine intake and status within populations is an area of concern and research focus. This paper will review recently published studies that focus on the re-emerging issue of iodine deficiency as a global concern and declining intake among populations in developed countries. Historically, the implementation of salt-iodization programs worldwide has reduced the incidence of iodine deficiency, but 30% of the world’s population is still at risk. Iodine nutrition is a growing issue within industrialized countries including the U.S. as a result of declining iodine intake, in part due to changing dietary patterns and food manufacturing practices. Few countries mandate universal salt iodization policies, and differing agriculture and industry practices and regulations among countries have resulted in inconsistencies in supplementation practices. In the U.S., in spite of salt-iodization policies, mild-to-moderate iodine deficiency is common and appears to be increasing. European countries with the highest incidence of deficiency lack iodization programs. Monitoring the iodine status of at-risk populations and, when appropriate, public health initiatives, appear to be warranted.

## 1. Introduction

Iodine, a trace element, is an essential component of thyroid hormones thyroxine (T4) and triiodothyronine (T3), which are critical for liver, kidney, muscle, brain and central nervous system function [1]. Iodine regulates overall metabolism [1] and plays a critical role in fetal and child neurodevelopment, organ and tissue function. A healthy adult body contains 15–20 mg of iodine, of which 70–80% is in the thyroid gland [2]. For the developing fetus, deficiency is one of the greatest causes of preventable intellectual disability [3], thus iodine status of pregnant and women of reproductive age is a recognized international concern.

Iodine intake occurs from several dietary sources, but until the fortification of table salt began in the 1920′s deficiency was present throughout the U.S., especially in regions where the topsoil is depleted of iodine [4]. Iodine status in the U.S. has not been considered to be a significant issue since the 1940′s, unlike many other parts of the world [5]. However, data from recent decades suggests that iodine status has been falling in the U.S. [6]. After 1990, salt iodization programs were introduced and have globally reduced the prevalence of iodine deficiency in many populations [7] although currently 30% of the world’s population is at risk of deficiency [5]. A reduction in iodine status has recently been observed in industrialized countries such as the U.S., UK and Australia, which may be due to a change in dietary patterns, food preparation and agricultural practices [4]. Woodside and Mullan have recently published a comprehensive discussion of how changes in food preparation, dietary intake patterns and agricultural practices may have reduced iodine intake [8]. The decline in iodine status among women of reproductive age in these countries is of particular concern [9]. Therefore, the objective of this scoping review is to examine research studies and review papers that provide perspective on iodine as an important, understudied nutrient and focus on the re-emerging issue of iodine deficiency as a global concern as well as the surprising decrease in iodine consumption among populations in developed countries. This review addresses the topics of iodine distribution and metabolism, methods of assessment with a focus on urinary iodine concentration (UIC), Dietary Reference Intakes (DRI), deficiency (populations at risk and associated consequences), excessive intake, changing dietary patterns, food sources of iodine, labeling and regulation, supplementation, and global iodine nutrition.

## 2. Distribution of Iodine in the Environment

Iodine is the heaviest stable halogen element and primarily exists in nature as iodide (I-), the form typically used to produce supplements and iodized table salt in the form of potassium iodide (KI) [2]. It can also be found naturally as iodate (IO_3_-), another form used to fortify table salt as potassium iodate (KIO_3_) [2]. Iodide is naturally in soil and seawater, which affects the iodine content of produce [7]. However, in many regions surface soils are depleted of iodide. Since iodide is found in seawater, it volatizes into the atmosphere and can return to the soil [10]. In non-coastal regions this cycle is incomplete and as a consequence plant foods and drinking water are depleted [10]. Therefore, historically iodine deficiency was seen in populations from inland regions (central Asia and Africa, central and eastern Europe, the central U.S.), mountainous areas (Alps, Andes, Atlas, Himalayas) and those with frequent flooding (Southeast Asia) [10]; consequently, these populations depend on the availability of iodized salt so the geographic distribution of deficiency may be more homogeneous.

## 3. Iodine Metabolism

In the human gut iodine is metabolized to iodide and in healthy adults > 90% is absorbed [10]. Once in circulation, it mainly accumulates in the thyroid gland or is excreted by the kidney, with small amounts accumulating in the salivary glands, mammary gland or choroid plexus [1]. Clearance by the kidney is constant, while uptake by the thyroid is dependent upon iodine status and intake. When iodine supply is sufficient, uptake by the thyroid may be ≤10%, whereas it can exceed 80% due to chronic deficiency [2]. After active transport into the thyroid, iodide is stored in the thyroglobulin (Tg) protein before undergoing conversion into T3 and T4. The hormones T3 and T4 enter circulation bound to carrier proteins and then target tissues; however, T3 is the primary physiologically active form and preferentially binds to its receptors [2]. Both free T3 and T4 are assessed in serum by immunoassay [1]. However, serum thyroid stimulating hormone (TSH) is the best marker of individual thyroid status and is used clinically for this purpose. The hormones T3 and T4 do not change sufficiently as a result of iodine deficiency to be clinically useful [1].

Over 90% of ingested iodine is excreted in the urine by the kidneys, which is why measurement of UIC is considered one of the best biomarkers of recent dietary iodine intake [1,11]. The hormone TSH, secreted by the pituitary gland, is the main regulator of T3 and T4 metabolism through a negative feedback loop regulated by thyroid hormones and modulated by TSH-releasing hormone [2]. In addition to signaling degradation of Tg and release of T3 and T4 into the circulation, TSH also increases iodine uptake; therefore, an elevated TSH concentration is generally a symptom of hypothyroidism, while low TSH levels indicate hyperthyroidism [2].

## 4. Methods for Assessing Iodine Status

The four main methods used to assess iodine status at a population level include UIC, serum Tg, serum TSH, and thyroid size [12]. Because iodide in the blood is either taken up by the thyroid and converted into thyroid hormones or excreted in the urine, serum levels of iodide are not used as indicators of iodine status among individuals [13]. Urinary iodine concentrations are sensitive markers reflective of recent dietary intake (days), whereas Tg represents an intermediate response (weeks to months), and changes in thyroid size reflect long term iodine nutrition [2,12]. Among individuals, UIC varies greatly on a daily basis, but this variation tends to even out at the population level which is why median UIC is the preferred method to monitor iodine status among populations, as discussed below [13]. Methods of iodine status assessment are not equally applicable in all population subgroups. For example, in older children and adults with iodine deficiency, TSH values do not differ from those with adequate iodine intake as long as daily intake exceeds 50 µg/day–below this level of intake thyroid iodine storage is low leading to hypothyroidism [12]. Although serum TSH is the best indicator of thyroid function, it is not a sensitive indicator of iodine status in adults and only recommended for neonates, as a supplement to UIC screening [14]. Similarly, thyroid hormone concentrations are not recommended for use as indicators of iodine status, as T3 and T4 levels in iodine-deficient populations often remain in the normal range [2]. Serum Tg is a non-specific marker of thyroid activity and has traditionally been used as a monitoring tool in thyroid cancer patients [14]; however, Tg assessed in a dried blood sample has been used as a marker of iodine status in children [15]. Additionally, a recent clinical study demonstrated the potential utility of Tg as a marker of iodine status in adults as it is associated with UIC concentration during iodine supplementation [16]. Thyroid enlargement and development of goiter as a result of long term iodine deficiency can develop in infants, children and adults when adaptation to chronic iodine deficiency occurs. In its early stages, goiter appears as diffuse thyroid enlargement, and as it progresses nodules from accumulation of new thyroid follicles, primarily in adults, may occur [12]. Thyroid size or volume may be used as methods to determine the severity of iodine deficiency. Both are inversely correlated with the magnitude of deficiency when assessing population data [12].

## 5. Urinary Iodine Concentration

Measurement of the median UIC from spot urine samples of a population is a preferred method to determine iodine status at the population level, and is typically expressed as µg/L [14]. Because of considerable daily variation in iodine excretion, the use of UIC from spot urine as a diagnostic tool for iodine status on an individual level is not recommended [14]. However, an individual’s daily iodine intake may be calculated using an equation that incorporates UIC and is based on several assumptions [17]. Urinary iodine excretion can be determined over a 24 h period (µg/day) from the urine collected at a specific time, or in relation to creatinine excretion (µg/g creatinine) [17]. Individual iodine status is most accurately estimated by the level excreted in the urine over a 24 h period, and recommendations suggest using multiple 24 h collections for reliability [18]. In population assessment, 24 h urine collection is not typically feasible and therefore spot urine levels expressed as the median in µg/L, are used [2]. Generally, using a large number of spot samples will account for individual variation in urine concentration and the median UIC will correlate with that from 24 h samples [2].

A median UIC of 100–199 µg/L is considered adequate iodine status for non-pregnant, non-lactating adults and children ≥ 6 years of age, whereas a median UIC of 150–249 µg/L is adequate for pregnant women, and concentrations ≥ 100 µg/L are adequate for lactating women and children < 2 years of age [12,19]. Severe iodine deficiency is defined as UIC concentrations < 20 µg/L [7,19]. The World Health Organization (WHO) defines adequate iodine intake in a non-pregnant population based on a median UIC of 100–199 µg/L because a UIC of 100 µg/L typically represents an iodine intake of 150 µg/day [12]. National Health and Nutrition Examination Survey (NHANES) data from 2011–2014 indicate the median UIC of the U.S. population age 6 years and older is 133 µg/L [9]. Median UIC is inversely correlated with characteristics of iodine deficiency, as the incidence of nodular goiter increases as median UIC declines [20].

## 6. Recommended Intakes of Iodine

To maintain homeostasis and hormone synthesis, the thyroid absorbs 50–60 µg/day of iodine when supply is sufficient [2]. The daily recommended dietary allowance (RDA) established by the Institute of Medicine (IOM) is 90 μg for children 1–8 years old, 120 μg for children 9–13 years old, 150 μg for males and most females ages 14 and older, 220 μg for pregnant women and 290 μg for lactating women [17]. The daily Tolerable Upper Intake Level (UL) for individuals over age 18 not receiving iodine for medical reasons is 1100 μg [17]. The U.S. Food and Drug Administration (FDA) does not require naturally occurring iodine contained in a food item to be included on the nutrition facts panel, but a food product fortified with iodine must include the % Daily Value (DV) on the label [21].

## 7. Iodine Deficiency

Iodine is one of the most common nutrient deficiencies and is estimated to affect 35–45% of the world’s population. Iodine deficiency is the most common cause of goiter and worldwide is estimated to affect 2.2 billion people, however not all goiters are the result of an iodine deficiency. The incidence of goiter is based on the degree of iodine deficiency. With mild iodine deficiency, the incidence of goiter is 5% to 20%. With moderate deficiency, the incidence is 20% to 30%, and with severe iodine deficiency, the incidence is greater than 30% [22]. In the U.S. in 2011–2012, 38% of the population had a UIC of <100 and were therefore classified as iodine deficient [23]. In the total U.S. military population, from 1997–2015, the incidence of clinically diagnosed iodine deficiency substantially increased in males but overall was more common in females and racial minorities [24]. Hypothyroidism, a symptom of severe iodine deficiency, is present in approximately 5% of the U.S. population. We are not aware of any recently published data on the incidence of iodine deficiency in the U.S. general population. The most recent NHANES data collected in 2011–2014, indicated that certain sub-groups of the population were at greater risk of iodine deficiency [9,25].

Recently it was reported that 23% of a sample of pregnant women in Michigan had inadequate intake of iodine [26]. As of 2001, the populations of most European countries exhibited mild to moderate deficiency, as 17% of the population were at risk of iodine deficiency disorders (IDD) [27]. In the United Kingdom a recent survey conducted in 2016–2018 found that 17% of women between the ages of 16–49, an especially vulnerable population, were iodine deficient [8]. In a recent study of pregnant women in Norway at 18 weeks of gestation median UIC was 94 µg/L indicating mild-to-moderate deficiency and lower iodine availability were associated with lower TSH [28]. In South East Asia and the Eastern Mediterranean IDD is estimated to be present in 36% and 43% of the general population, respectively [27]. The frequency and severity of IDD is determined by the severity of iodine deficiency. Individuals with mild deficiency risk thyroid gland enlargement, benign thyroid nodule formation with or without goiter, and formation of an endemic goiter, which is a goiter found among individuals from a specific geographic area [4,20]. In adults, severe deficiency presents as hypothyroidism, goiter, mental disability and decreased fertility. In children, goiter, intellectual/physical developmental impairment, deafness and cretinism can occur [1,2,29].

The thyroid gland adapts to iodine deficiency by increased absorption of iodide and increased intrathyroidal metabolism as a result of elevated TSH levels, leading to enlargement of the gland and goiter development [29]. This occurs at an accelerated rate in the pediatric population with severe deficiencies, and endemic cretinism is the most serious complication of iodine deficiency which is characterized by a combination of intellectual disability with a neurological syndrome (neurological cretinism) or hypothyroidism (myxedematous cretinism) or both [27,29]. The diagnosis of goiter is most commonly determined by total thyroid volume using ultrasound technology, and nodular goiter is characterized by intrathyroidal lesions or follicles that fuse and become encapsulated [2,20]. Multinodular goiter can be classified as euthyroid and toxic depending on the clinical presentation, epidemiology and molecular pathology [30]. Mutations that facilitate growth of thyroid cells can lead to thyroid carcinoma in multinodular goiter [30]. There are certain foods that contribute to the cause of IDD as discussed below.

## 8. Populations at Risk of Iodine Deficiency

Women of reproductive age are one subgroup of the U.S. population in which the prevalence of iodine deficiency is increasing [12]. NHANES data from 2009–2012 indicate women aged 20–39 years and young adult non-Hispanic black individuals are demographic groups with higher prevalence of median UIC < 50 µg/L [6]. The percentage of all individuals falling within this category decreased from the 2001–2004 survey period to the 2005–2008 survey period, but increased again by the 2009–2012 period [6]. Earlier U.S. data from 1988–1994 also showed an increase in the proportion of the population classified within this category from the 1971–1974 survey period [25]. NHANES data from 2011–2014 indicate that women of reproductive age (15–44 years) have an overall median UIC of 110 µg/L with a linear decline as age increases; the 40–44 year age group at 91 µg/L [9]. Non-Hispanic Asian women of reproductive age had the lowest median UIC at 81 µg/L, indicating mild iodine deficiency. Data collected between 2008–2015 from a cohort of pregnant women in Michigan indicate that although median UIC in each trimester (171 µg/L, 181 µg/L, 179 µg/L) was above the recommended cutoff of 150 µg/L for pregnancy, 23% of women were still consuming inadequate levels of iodine [26]. Recent NHANES data from 2011–2014 indicate that only 15% of women of reproductive age use an iodine-containing dietary supplement, and those in the non-Hispanic black demographic were even less likely to use any supplement [31]. The increases in the rise of iodine deficiency in the US may be related to the reduced iodine content of the food supply as discussed below.

In pregnant women, severe iodine deficiency presents as hypothyroxinemia, elevated serum TSH, enlarged thyroid and goiter [32]. When iodine deficiency is present in the developing fetus, it can lead to abortion, stillbirth and increased perinatal and neonatal mortality or congenital hypothyroidism [29]. From a public health perspective, pregnant women, fetuses, neonates and infants are the most vulnerable groups because of the irreversible effects of iodine deficiency disorders (IDD) which leads to brain damage and intellectual development disorders [29]. Damage to reproductive function, the developing fetal brain and infant are the most severe consequences of IDD [32], and low placental weight and reduced newborn head circumference have been linked to mothers with lower iodine status [33]. Iodine intake requirements for pregnant women are increased due to the needs of the developing fetus and to address the higher renal iodine losses seen in pregnant women. Requirements for lactating mothers are even higher as they lose an average of 114 µg/day in breast milk to support the growing infant [32]. Hyperthyroidism and multinodular goiter may develop from iodine deficiency in mothers even after lactation terminates due to the amplified iodine losses incurred [32]. Only about 18% of pregnant women and 19% of lactating women in the U.S. use an iodine-containing supplement according to 2011–2014 NHANES data [31].

## 9. Adverse Consequences of Excess Iodine Intake

Excessive iodine status is rare and difficult to define, but a median UIC of > 299 µg/L indicates possible excess [1]. In the U.S. the IOM has set the UL for iodine intake at 1100 µg/day for adult men and women, which includes pregnant and lactating women 19 years and older [17], although the consequences of excess intake are far less detrimental than too little [1]. Excessive iodine intake may lead to hyperthyroidism, autoimmune thyroid disease, and papillary cancer–a type of thyroid cancer [32]. In healthy adults, high levels can reduce thyroid hormone production, leading to higher TSH stimulation, causing hypothyroidism and eventually thyroid growth and development of diffuse goiters [1,2,20]. In populations that are chronically iodine deficient, sudden increase and excess iodine intake leads to iodine-induced hyperthyroidism, which primarily occurs in older individuals with nodular goiter [2]. Excessive intake has been observed in Japan, due to excessive seaweed consumption, and Chile, a largely coastal country, due to oversufficient salt iodization, extensive use in water purification, and broad availability of environmental iodine [1]. Although a limited problem, high iodine levels have adverse health effects, thus ensuring proper intake is vital.

## 10. Dietary Sources and Recent Changes in Iodine Content of Foods

Changing dietary patterns and food processing techniques in the U.S. may be contributing to the increase in iodine deficiency observed in recent decades. Foods contributing to the highest dietary intake of iodine in the U.S. are bread, dairy products and iodized salt [9,34]. Other foods high in iodine are eggs, fish and seaweed [35]. A recent report that provided detailed information on iodine in the U.S. diet observed considerable variability in some food groups such as species of seafood, commercial breads, and milk [35]. In this report, over 400 U.S. food samples from various regions were collected and analyzed by the FDA and U.S. Department of Agriculture (USDA) to provide a comprehensive source of data on the iodine content in food [35]. The focus of analysis was on seafood, dairy/eggs, bread and baked goods, the primary iodine contributors to the U.S. diet [35], as very few foods contain high amounts of natural iodine. For example, dairy/eggs were foods reported to have the highest iodine content, but the median content of the group was only 42 µg per 100 g sample. Plain, nonfat Greek yogurt had an average iodine content of ~50 µg per 100 g sample. That would equate to roughly 75 µg in a typical 150 g single portion container of yogurt, which is only half of the daily recommended intake for adult males and non-pregnant females. Likewise, 100 g of hard-boiled eggs contains roughly 50 µg of iodine, which would only amount to 25 µg in a single 50 g hard-boiled egg. As such, portion size must be considered when evaluating the actual contribution of foods naturally high in iodine to dietary intake. On the other hand, fortified foods can contribute much more iodine in smaller portions, as is observed with bread samples made with iodate dough conditioners [35], although commercial bakeries are more commonly discontinuing their use [34,35]. Data from 11 different hamburger bun samples (50 g serving) resulted on average with 598 µg per bun of iodine, almost four times the dietary recommendation for adults. These samples were collected from up to 24 sampling locations and 4–6 different U.S. regions and chemically analyzed at validated laboratories (USDA samples) or in Kansas City (FDA samples); however, the dates of analysis were not specified and may not reflect current food processing or agricultural practices [35].

Changes in agriculture and industry practices in the U.S. and other industrialized countries may be contributing to the decline of iodine content in the food supply [8]. Reduced use of iodate dough conditioners may have affected iodine content in store-bought breads and baked goods, while reduced use of iodine supplemented feed for livestock may be contributing to lower iodine content of dairy milk, meat, and eggs [12,34]. Reduced use of iodophors as sanitizing agents in milk processing may influence the iodine content in dairy products [35]. In addition, iodine-containing compounds used in fertilizers and irrigation affect vegetation consumed by livestock in feed [10]. Organic food practices in agriculture appear to decrease the iodine content of animal feed, as iodine levels in non-organic supplemental feed have been found to be as much as 10x higher than those in forage feed [8,36]. Organic farming practices may significantly reduce the iodine content of organic milk, as discussed below.

Use of iodized salt is an effective public health measure to ensure adequate iodine intake [12]; however, up to 20% of iodine in salt may be lost during processing, and another 20% lost during food preparation [37]. Furthermore, and of concern, is that only 53% of table salt sold in U.S. retail stores is iodized [38]. Lower dietary salt use at the table and in cooking due to public health messages linking high sodium intake to hypertension are a contributing factor to reduced iodized salt intake, in addition to use of non-iodized salt in processed and restaurant foods [6,35] and higher use of sea salt, which is naturally low in iodine, compared to iodized salt in cooking [35].

Other consumer practices that may contribute to recent declines in dietary intake of iodine include veganism and some forms of vegetarianism [39,40,41] which have substantially increased in popularity in industrialized countries in recent years [41]. For example, in Britain the number of vegans quadrupled to 600,000 from 2014 to 2018 and they have lower iodine levels than the general population [8,23]. Furthermore, plant-based food alternatives such as oat milk are not typically fortified with iodine, and consumption of these foods have nearly doubled between 2014 and 2017 in the UK [41]. In the U.S, plant-based food sales rose 8% in 2017, and plant-based dairy alternatives were predicted to reflect 40% of so called dairy beverage sales in 2018 [42].

Further contributing to declining iodine status is the increased avoidance of dairy intake, along with the emergence of plant-based alternative milk sources on the market [43]. Some foods, such as soy-based dairy alternatives interfere with iodine absorption [44], and a number of milk alternative products have been shown to contain much less iodine than cow’s milk [45]. Organic farming practices in the UK contribute to lower iodine content of some foods compared to conventional practices, as organic milk is 25–40% lower in iodine [8]. Data from other industrialized countries also reveal lower iodine concentration in organic vs. conventional milk [46]. Strict requirements on limiting mineral supplementation of feed, regulations on annual days that cows must be out to pasture, higher dietary goitrogen content of a 60% forage diet, and the preference of clover pasture over nitrogen fertilizer may be contributors to the lower iodine content in organic milk [8,46].

Dietary factors can contribute to the cause of IDD, as certain foods are sources of natural goitrogens which interfere with thyroid metabolism. Cruciferous vegetables (i.e., broccoli, cabbage, kale, cauliflower) contain glucosinolates that have metabolites (thiocyanate and isothiocyanate) known to compete with thyroid iodine uptake [10]. The metabolism of cyanogenic glucosides leads to production of cyanide and subsequently thiocyanate, and this group of goitrogens are found in certain vegetables such as cassava, sweet potatoes, maize, lima beans, bamboo shoots, linseed and sorghum [10,29]. Cassava is a staple in many developing countries and has been linked to the etiology of endemic goiter in Africa and Malaysia [29]. It contains linamarin, which will produce thiocyanate if the vegetable is not soaked or cooked properly before consuming [2]. Additionally goitrogenic flavanoids in soy and millet may interfere with enzymatic activity involved in iodine metabolism [10]. Cooking prior to consumption can minimize goitrogenic effects [2].

## 11. Labeling and Regulation of Iodine in Foods and Dietary Supplements

In the U.S., iodine fortification of salt, infant formula and food are regulated by the FDA, but dietary supplements, including prescription prenatal vitamins, are regulated differently than conventional foods and drugs and iodine is not a required component of prenatal dietary supplements [21,47]. However, the FDA does mandate the requirement of Nutrition Facts labels on both food and supplements. As mentioned previously, it is optional for manufacturers to list iodine % DV on the Nutrition Facts label for food items that naturally contain it [21]. In the case of supplements, the FDA does not regulate ingredient standards, but under the Dietary Supplement Health and Education Act of 1994, manufacturers must list ingredient information on the label; thus if iodine is added to a supplement, the label would indicate as such [48]. Iodine compounds that are considered Generally Recognized as Safe by the FDA include Cuprous iodide (CuI) and KI, which are additives to table salt; and KIO_3_ and Calcium iodate Ca(IO_3_)_2_ used as dough strengtheners in bread production [21]. Table salt that is iodized must indicate so on the label, and likewise if it has not been fortified, a statement must be present on the label [21]. Similar to the FDA, the Codex Alimentarius established by the United Nations Food and Agriculture Organization and WHO sets forth international codes of practice and food standards (to include labeling and voluntary/mandatory addition of nutrients to food) for the purpose of consumer protection and health as well as fair food trade practices [49]. However, a government’s use of Codex standards is voluntary [21].

## 12. Iodine Supplementation

Iodine supplementation is an effective way to reduce population iodine deficiency, but precautions must be taken to prevent excessive intake. Iodine intake should be increased to the point at which IDD is prevented, but not higher [50]. Bioavailability of iodine from food varies and is difficult to assess, and interactions between different foods within the food matrix are not well characterized [37]. The most practical and cost-effective way to provide iodine supplementation to deficient populations is with iodized salt, as advocated by several international organizations such as WHO, United Nations Children’s Fund, and International Council for Control of Iodine Deficiency Disorders, but other approaches include consumption of iodized water, iodized oil, and iodine tablets [1,10]. The quantity and type of iodine used for salt fortification varies by region but typically occurs within the range of 20–40 mg iodine/kg salt. The forms that may be used in fortification globally are either KIO_3_ which is more stable, or KI, which has a higher iodine content and solubility [1,10], or sodium iodide (NaI) [51]. Unlike iodization of salt and water which can reach a larger proportion of the population, supplementation with iodized oil or tablets is more appropriate on an individual basis and can increase iodine status rapidly [1], especially in regions in which salt iodization is not feasible or cannot be implemented in the short-term [10].

## 13. Global Iodine Nutrition

Universal salt iodization refers to iodization of all salt for human (household and industry) and livestock consumption, but is often difficult to achieve because of poor implementation by the food and agricultural industries [10]. Global salt iodization programs benefit not only individuals but also the community’s socioeconomic status. Negative effects of IDD include lower school performance, lower productivity of the workforce, and higher healthcare costs [1]. Prior to iodization programs, the annual cost attributed to losses from iodine deficiency in the developing world compared to the annual cost of salt iodization were estimated to have benefit:cost ratio of 70:1 [10].

Iodine supplementation programs have been initiated worldwide [50], however in Europe, legislation and regulation of iodine fortification varies by country so that regions with the highest incidence of deficiency are not necessarily taking the appropriate actions [51]. Among WHO countries, the European region has the lowest extent of salt iodization, even compared to countries with lower socioeconomic status [51]. Part of the contributing causes to low coverage are social and political changes affecting the salt iodization process in addition to increasing food globalization [51]. In the UK especially, decline in iodine status among females (teenagers, pregnant women and women 16–49 years) is attributed not only to lack of iodine fortification programs, but to changes in farming practices, dietary preference, and public health priorities [8]. Surveillance of program implementation and monitoring of population iodine status is key not only to prevent complacency and regression of a population’s progress [1,51] but also to ensure consistency and avoid excessive intake in attempts to overcorrect deficiency [50].

## 14. Conclusions

Although progress has been made in many parts of the world that have improved iodine status, there are still many areas and specific populations in need of iodine supplementation, including the U.S. and European countries, remote regions which cannot be reached through iodization programs, and, globally, women of reproductive age. Assessment of iodine status with UIC measurement is the optimal method to assess intake, and salt fortification is the principle measure to provide population-wide supplementation. Of considerable concern are changes in industrial and agricultural practices and dietary patterns in industrialized countries such as the U.S. and UK [8,41,45,46] that seem to be contributing to the declining iodine status observed in the U.S. after the 1970’s. Current trends in the U.S. and other countries should be assessed using nationally representative population samples such as NHANES. Changes in U.S. regulations and the efforts of international organizations to ensure use of iodized salt throughout the world are essential, as is surveillance of those programs to ensure adequate and safe levels of iodine intake. Additionally, of critical importance are educational campaigns especially for populations at high risk of iodine deficiency. Future research should include continuous monitoring of iodine status among the U.S. and international populations in addition to tracking intake of traditional iodine-containing foods and iodine content over time. Research to monitor changes in the iodine content of iodine-containing foods is also essential given ongoing changes in agricultural and food preparation practices. Monitoring of public health initiatives that aim to increase iodine intake should also be a priority. Lastly, further research is necessary to determine whether revised or new regulatory actions should be considered in an effort to increase iodine intake.

## Data Availability

Not Applicable.
